# Interleukin-6 and interleukin-6 receptor secretion by chronic lymphatic leukaemia and normal B lymphocytes: effect of PMA and PWM

**DOI:** 10.1080/09629359791857

**Published:** 1997-04

**Authors:** I. Drucker, A. Klajman, M. Revel, Y. Manor, S. Ben-Efraim, D. Novick

**Affiliations:** 1 Laboratory for Clinical Immunology Meir Hospital Kfar-Saba 44281 Israel; 2 Department of Molecular Genetics and Virology The Weizmann Institute of Science Rehovot Israel; 3 Department of Hematology Meir Hospital Kfar-Saba Israel; 4 Department of Human Microbiology Sackler Faculty of Medicine Tel-Aviv University Tel-Aviv Israel

## Abstract

Interleukin-6 (IL-6) and soluble interleukin-6 receptor (sIL-6R) were detected in supernatants of cultures of B chronic lymphatic leukaemia (CLL) lymphocytes. Phorbol-12-myristate 13 acetate (PMA) caused a decrease in the levels of IL-6 in 14 out of 16 cultures and an increase in levels of sIL6R in all 15 cases. The effect of pokeweed mitogen (PWM) was variable and not significant. The levels of IL-6 were below the detection limit (60 pg/ml) in sera of 13 CLL patients whereas sIL-6R was detected (13 ng/ml to 97 ng/ml) in the 13 sera. IL6 was not detected in cultures of unstimulated or stimulated with PMA or PWM normal human B cells. Levels of sIL-6R were minimal in cultures of normal B lymphocytes and were increased in PMA stimulated cultures. The results are consistent with the view that B-CLL cells produce spontaneously IL-6 which could act in an autocrine fashion to cause shedding of surface IL-6R and account for the correlation found between serum levels of sIL-6R and B-CLL lymphocyte numbers. The fall in levels of IL-6 in PMA stimulated CLL cultures might express masking or degradation of IL-6 after combination with the receptor.


Mediators of Inflammation, 6, 147–153 (1997)

### Interleukin-6 and interleukin-6 receptor secretion by chronic lymphatic leukaemia and normal B lymphocytes: effect of PMA and PWM

I. Drucker,[1] A. Klajman,[1,CA] M. Revel,[2] Y. Manor,[3] S. Ben-Efraim[4] and D. Novick[2]

[1]Laboratory for Clinical Immunology, Meir Hospital, Kfar-Saba 44281; [2]Department of Molecular Genetics and Virology, The Weizmann Institute of Science, Rehovot; [3]Department of Hematology, Meir Hospital, Kfar-Saba; [4]Department of Human Microbiology, Sackler Faculty of Medicine, Tel-Aviv University, Tel-Aviv, Israel

[CA]Corresponding Author
Fax: (+972) 9 9576 139

**INTERLEUKIN-6 (IL-6) and soluble interleukin-6 receptor (sIL-6R) were detected in supernatants of cultures of B chronic lymphatic leukaemia (CLL) lymphocytes. Phorbol-12-myristate 13 acetate (PMA) caused a decrease in the levels of IL-6 in 14 out of 16 cultures and an increase in levels of sIL-6R in all 15 cases. The effect of pokeweed mitogen (PWM) was variable and not significant. The levels of IL-6 were below the detection limit (60 pg/ml) in sera of 13 CLL patients whereas sIL-6R was detected (13 ng/ml to 97 ng/ml) in the 13 sera. IL-6 was not detected in cultures of unstimulated or stimulated with PMA or PWM normal human B cells. Levels of sIL-6R were minimal in cultures of normal B lymphocytes and were increased in PMA stimulated cultures. The results are consistent with the view that B-CLL cells produce spontaneously IL-6 which could act in an autocrine fashion to cause shedding of surface IL-6R and account for the correlation found between serum levels of sIL-6R and B-CLL lymphocyte numbers. The fall in levels of IL-6 in PMA stimulated CLL cultures might express masking or degradation of IL-6 after combination with the receptor.**

**Key words:** B lymphocytes, CLL, IL-6, PMA, PWM, sIL-6R.

### Introduction

Interleukin-6 (IL-6) is a pleiotropic cytokine produced by adult T[1] and B[2] lymphocytes, fetal mononuclear cells,[3] macrophages,[4] fibroblasts,[5] endothelial cells and chondrocytes.[5] IL-6 is involved in growth of myeloma cells,[6] induction of IL-2 and IL-2 receptor expression,[7] maturation of megakaryocytes,[8] and production of acute protein in liver cells.[5] IL-6 was reported to induce immunoglobulin secretion by activated B cells. It acts on various cells by binding to IL-6 specific receptor and by activation of the associated signal transducer gp130.[9] Various soluble cytokine receptors were recently identified: IL-6,[10] IFN-γ,[11] TNF-α[12] and IL-1α.[13] Antibodies to human IL-6 receptor (IL-6R) were prepared,[14] thus enabling the study of the expression of IL-6R on the cell surface and the levels of soluble IL-6R (sIL-6R) in human sera.

B-cell chronic lymphatic leukaemia (B-CLL) is a malignant clonal proliferation of apparently mature $CD5^{+}$B lymphocytes. The number of pathological B cells varies from a few thousand to more than one hundred thousand per 1 μl.

The present study was intended to determine IL-6 and sIL-6R levels in supernatants of short-term cultures of CLL B lymphocytes and normal peripheral blood human B lymphocytes (NB) in non-stimulated cultures and culture stimulated with either pokeweed mitogen (PWM) or phorbol 12-myristate 13 acetate (PMA). Concomitantly the levels of IL-6 and sIL-6R in sera of CLL patients were determined.

### Materials and Methods

### Samples

Sterilized heparinized peripheral blood was obtained after consent from untreated B-CLL patients attending the outpatient haematology clinic at Meir General Hospital, Kfar-Saba, Israel and from normal volunteers. Freshly obtained or cryopreserved lymphocytes were studied. The stage of B-CLL varied from 0 to 3 according to Rai classification.[15]

### Separation of lymphocytes

Peripheral blood mononuclear cells (PBMC) were separated from peripheral blood by Ficoll-hypaque density centrifugation.[16]

© 1997 Rapid Science Publishers

### Positive selection of B cells

PBMC from healthy subjects were treated with Dynabeads M450 coated with anti-CD19 antibodies (Dynal AS, Oslo, Norway), as recommended by the manufacturers. The $CD19^+$ cells were released from the beads by 24 h incubation at 37°C and removed by the Dynal Magnetic Concentrator. The B-cell percentage was determined by staining the cells with fluoresceinated antitotal Ig.

### Negative selection of B cells

PBMC were incubated on plastic plates for 1 h at 37°C to remove the adherent cells (monocytes). The nonadherent cells were treated with Dynabeads coated with anti-CD2 antibodies (Dynal AS) as recommended by the manufacturers. Non-adherent cells were removed and consisted of highly purified B cells.

### Culture of B-CLL or normal B lymphocytes

B-CLL lymphocytes were used either as PBMC after Ficoll-hypaque separation or as purified B cells obtained after negative selection. Highly purified normal B lymphocytes were obtained after positive selection. The cells were cultured in 24-well tissue culture dishes (Nunc, Denmark) at a density of $5 \times 10^6$ cells/ml per well in RPMI-1640 medium supplemented with decomplemented pooled human serum, 10 mM Hepes, 1% glutamine and an antibiotic–antimitotic mixture (CM: complete medium). They were incubated at 37°C in a humid atmosphere and 5% $CO_2$ for a period of 3–7 days. The cells were cultured either in CM alone or in the presence of 10 µg/ml PWM or 10 ng/ml PMA. Subsequently, the cells were spun and the supernatants were collected and stored at −70°C until examination for IL-6 and sIL-6R secretion.

### Cell surface markers

The determination of cell surface markers was done as described previously[17] using anti-CD19 and anti-CD3 monoclonal antibodies (Dakopatts, Denmark) and polyclonal antitotal Ig. The second antibody was FITC conjugated rabbit anti-mouse immunoglobulin (Dakopatts, Denmark). The number of positively stained cells was counted either by FACS 440 (Becton-Dickinson, CA, USA) or under fluorescent epi-microscope.

### Determination of sIL-6R

The determination of sIL-6R levels in sera and in supernatants from B-CLL and normal B lymphocyte cultures was done by ELISA as described previously.[14] Microtitre plates (Dynatech Lab., Alexandria, Virginia, USA) or Nunc immunoplates were coated with anti-IL-6R monoclonal antibodies (immunoglobulin fraction, 120 µl/well, 20 µg/ml in PBS) and kept overnight at 4°C. The plates were washed in PBS with BSA (0.5%) and Tween-20 (0.05%) (blocking solution) and were incubated in the same blocking solution for at least 2 h at 37°C. The samples were diluted in the blocking solution containing also 0.65 M NaCl and 0.1% NP40 and were added to the cells (100 µl/well) and incubated for 4 h at 37°C. The plates were washed three times with PBS containing Tween-20 (0.05%) followed by addition of rabbit anti-sIL-6R serum (1:1000, 100 µl/well) and kept overnight at 4°C. The plates were washed three times and a conjugate of goat-antirabbit-immunoglobulin-horseradish-peroxidase (HRP, Bio-Makor, Israel), 100 µl/well was added at a dilution of 1:2000 and maintained for 2 h at room temperature. The plates were washed four times and the colour was developed by ABTS (2,2′-azino-bis-3-ethylbenzthiazoline-6-sulphonic acid, Sigma, USA) with $H_2O_2$ as substrate. The plates were read by an automatic ELISA reader.

### Determination of IL-6 levels

The ELISA was performed as described above for sIL-6R. Monoclonal anti-IL-6 antibody 34-1[18] was used for coating the microtitre plates and an affinity purified anti-IL-6 (100 ng/ml) was used for detection of IL-6.

### Monoclonal anti-sIL-6R antibodies

MoAb to sIL-6R were prepared as reported previously.[14] Monoclonal antibody number 17.66 gave the best results for ELISA and was used in all the experiments.

### Statistical analysis

Results are expressed as means ± SD. The Student's *t*-test was used to determine the statistical significance of the results.

### Results

### Levels of IL-6 and sIL-6R in supernatants of 15 cultures of B-CLL lymphocytes

The percentage of B cells at the onset of cultures varied from 82% to 90% and of $CD3^+$ cells from 7% to 18%. The cells were cultured for 3–7 days. In the supernatants from control (unstimulated) cultures the levels of IL-6 varied from 0.25 ng/ml to 33.7 ng/ml (Table 1). Stimulating the cells with PMA decreased significantly the levels of IL-6 in 12 out of 15 cases ($p = 0.0044$), induced no change in two cases and increased the IL-6 level in one case (Table 1). The levels of sIL-6R were increased in the presence of PMA in all 15 cases ($p = 0.0003$; Table 1). The effect of PWM on IL-6 levels was variable and statistically not significant: decrease in seven out of 15 supernatants, increase in three supernatants and no change in five supernatants (Tables 1 and 2). In six cases, the levels of IL-6 (Fig. 1) and of sIL-6R (Fig. 2) in unstimulated or cultures stimulated with either PMA or PWM, were compared after 3 and 7 days cultures. The levels of IL-6 did not change markedly in PWM or PMA stimulated cells by comparison with control unstimulated cultures. The levels of sIL-6R did not change markedly in PWM stimulated cultures by comparison with unstimulated cultures whereas PMA increased significantly the sIL-6R levels in five cultures ($p = 0.01$) and decreased in one supernatant.

**Table 1**. IL-6 and sIL-6R levels in supernatants of cultures of B-CLL B lymphocytes[a]

| Patient no. | Days in culture | Control cultures[b] (ng/ml) IL-6 | sIL-6R | PMA cultures[c] (ng/ml) IL-6 | sIL-6R | PWM cultures[d] IL-6 | sIL-6R |
|---|---|---|---|---|---|---|---|
| 1 | 3 | 33.7 | 0.31 | 5.9 | 0.46 | 29.3 | 0.18 |
| 2 | 5 | 21.8 | 0.22 | 1.6 | 0.82 | 8.3 | 0.20 |
| 3 | 5 | 6.7 | 0.06 | 2.6 | 0.49 | 9.8 | 0.1 |
| 4 | 5 | 0.7 | 0.23 | 0.25 | 1.8 | 0.4 | 0.47 |
| 5 | 6 | 22.9 | 0.06 | 7.64 | 0.22 | 18.6 | 0.22 |
| 6 | 3 | 0.25 | 1.05 | 2.84 | 1.24 | 33.44 | 0.75 |
| 7 | 3 | 1.39 | 0.16 | 0.25 | 0.40 | 1.48 | 0.29 |
| 8 | 5 | 6.93 | 0.21 | 1.86 | 0.34 | 6.22 | 0.31 |
| 9 | 5 | 20.44 | 1.13 | 13.81 | 1.63 | 27.91 | 0.96 |
| 10 | 5 | 1.54 | 0.29 | 0.25 | 1.86 | 1.52 | 0.30 |
| 11 | 5 | 11.7 | 0.06 | 0.40 | 1.19 | 6.78 | 0.02 |
| 12 | 5 | 13.4 | 0.14 | 1.47 | 1.23 | 6.88 | 0.21 |
| 13 | 5 | 11.5 | 0.24 | 1.19 | 1.27 | 8.16 | 0.42 |
| 14 | 5 | 0.25 | 0.06 | 0.25 | 0.48 | 0.25 | 0.27 |
| 15 | 3 | 0.25 | 0.17 | 0.25 | 0.46 | 0.25 | 0.27 |

Summary

| Cells[a] | Control cultures[b] (ng/ml[e]) IL-6 | sIL-6R | PMA cultures[c] (ng/ml[e]) IL-6 | sIL-6R | PWM cultures[d] (μg/ml[e]) IL-6 | sIL-6R |
|---|---|---|---|---|---|---|
| B-CLL | $10.2 \pm 10.4$ | $0.29 \pm 0.33$ | $2.7 \pm 3.8$[f] | $0.92 \pm 0.56$[f] | $10.6 \pm 11.27$ | $0.33 \pm 0.24$ |
| NB | 0 | $0.06 \pm 0.005$ | 0 | $0.52 \pm 0.31$ | 0 | $0.11 \pm 0.13$ |

[a]The cells were cultured ($5 \times 10^6$/ml) for 3 to 6 days.
[b]The cells were cultured in complete medium alone.
[c]PMA (10 ng/ml) or [d] PWM (10 μg/ml) were added to the culture. The level of sIL-6R in the medium was 0 45 ng/ml because of the presence of normal human serum and this amount was subtracted from the values obtained. [e]mean ± SD; [f] $p < 0.01$; B-CLL: 15 cases; NB: 5 cases.

**Table 2**. Effect of purification of B-CLL B cells stimulated with PMA or PWM on the levels of IL-6 and sIL-6R in supernatants of 5-day cultures[a]

| Case no. | Mitogen | PBMC % of B | % of T | IL-6 (ng/ml) | sIL-6R (ng/ml) | Purified B cells % of B | % of T | IL-6 (ng/ml) | sIL-6R (ng/ml) |
|---|---|---|---|---|---|---|---|---|---|
| 1 | Control | 82 | 18 | 9.79 | 0.15 | 93 | 3.5 | 8.53 | 0.31 |
| | PWM | | | 15.76 | 0.44 | | | 9.28 | 0.47 |
| | PMA | | | 5.55 | 0.57 | | | 3.14 | 0.71 |
| 2 | Control | 85 | 7 | 3.18 | 0.28 | 90 | 0 | 4.14 | 0.31 |
| | PWM | | | 5.99 | 0.24 | | | 4.72 | 0.72 |
| | PMA | | | 1.46 | 1.39 | | | 1.42 | 0.26 |
| 3 | Control | 90 | 12 | 9.02 | 0.16 | 99 | 3 | 2.7 | 0.31 |
| | PWM | | | 9.67 | 0.37 | | | 2.4 | 0.12 |
| | PMA | | | 3.89 | 0.65 | | | 0.89 | 0.78 |

[a]See Table 1 for details.

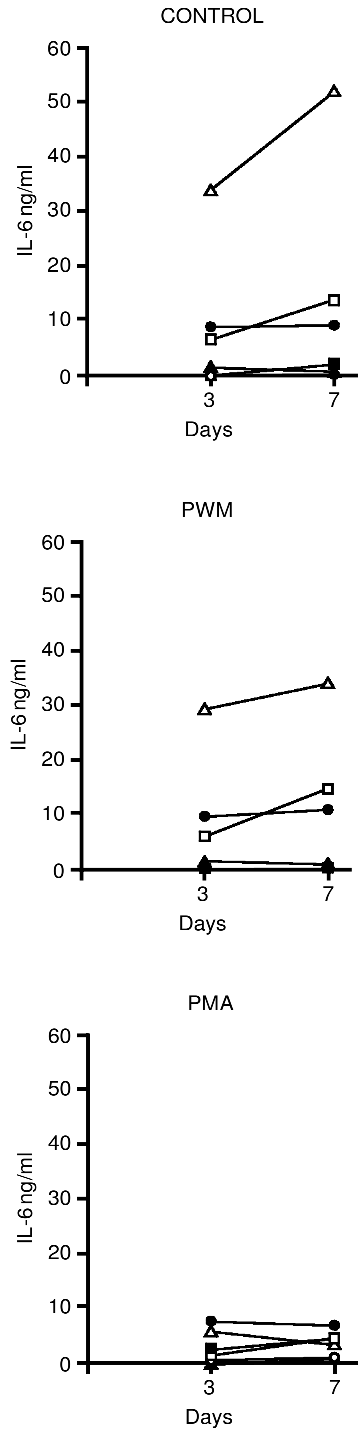

FIG. 1. The levels of IL-6 in supernatants from six B-CLL lymphocyte cultures after 3 and 7 days. The cells were either unstimulated (control) or stimulated with PMA (10 ng/ml) or PWM (10 μg/ml).

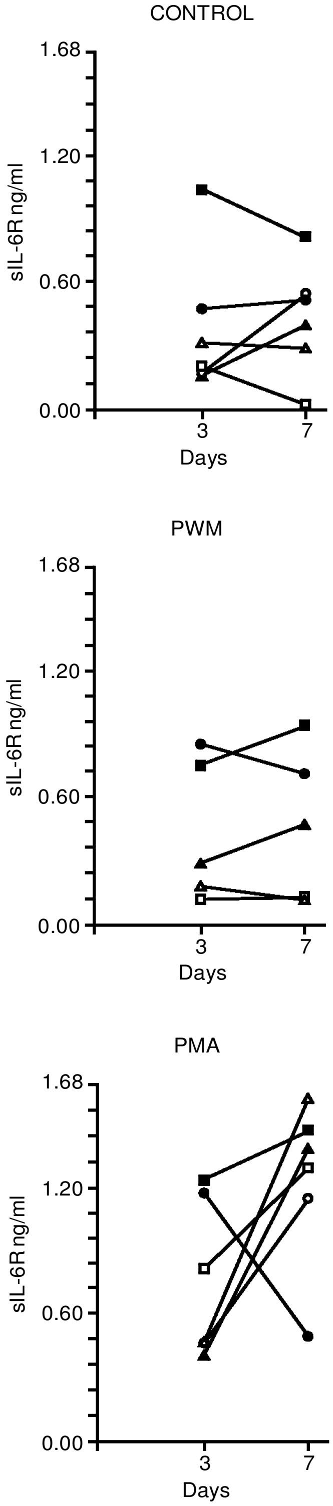

FIG. 2. The levels of sIL-6R in supernatants from six B-CLL lymphocyte cultures after 3 and 7 days. The cells were either unstimulated (control) or stimulated with PMA (10 ng/ml) or PWM (10 μg/ml).

Kinetic studies of IL-6 and sIL-6R secretion were done in cultures of one B-CLL subject (Fig. 3). Increase of IL-6 secretion was detected after 24 h with highest levels on the second day in unstimulated cultures. In the PMA stimulated culture the levels of IL-6 rose to a lesser extent than in the control (Fig. 3). A slight increase in the levels of sIL-6R was found already after 30 min of stimulation with PMA (Fig. 3) reaching a peak after 5 days. Low levels of sIL-6R were detected in the unstimulated culture after 2 h and decreased after 24 h of culture.

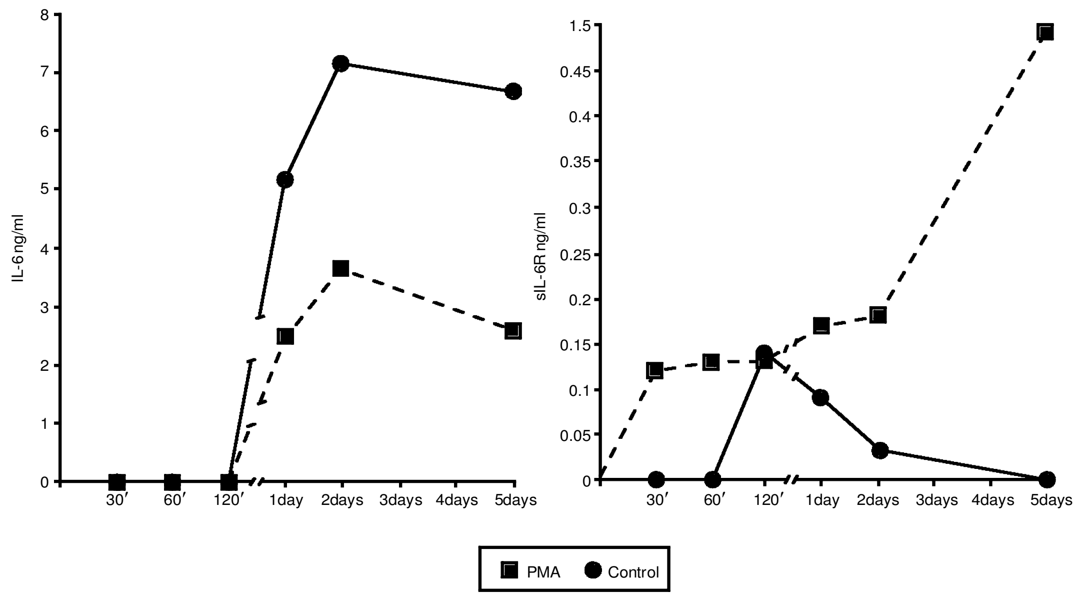

FIG. 3. A kinetic study of IL-6 and sIL-6R secretion from one case of B-CLL lymphocytes unstimulated or stimulated with PMA (10 μg/ml).

In three B-CLL patients the B cells were further purified by negative selection with CD2 coated beads and the percentage of B cells raised to 93%, 90% and 99% by comparison with 82%, 85% and 90% respectively in the non-enriched samples. The percentage of CD3 positive cells was 3.5%, 0% and 3% in the B purified samples versus 18%, 7% and 10% respectively in the non-enriched samples (Table 2). Purified B-CLL B cells and non-enriched PBMC were cultured for 5 days either in presence or absence of PMA or PWM (Table 2). Purified B cells paralleled the non-purified lymphocytes so far as levels of IL-6 and sIL-6R. The IL-6 levels decreased in all three purified B cultures stimulated with PMA (Table 2).

### Levels of IL-6 and sIL-6R in supernatants of normal B lymphocytes cultures

IL-6 was not detectable in 5-day cultures of unstimulated, PMA or PWM stimulated normal B cells. Only limit levels of sIL-6R were found in unstimulated or PWM stimulated cultures. In the presence of PMA, the sIL-6R levels increase to 0.52 ± 0.31 ng/ml (Table 1).

### IL-6 and sIL-6R levels in sera of B-CLL patients

The levels of IL-6 were below detection limits (< 60 pg/ml). In 13 sera of B-CLL patients the levels of sIL-6R were within the range of 13 ng/ml to 97 ng/ml: in eight cases the levels were above 40 ng/ml. Values of sIL-6R in 200 normal control sera varied between 20 ng/ml to 40 ng/ml as reported.[19] There was a good correlation between the number of B-CLL lymphocytes in the peripheral blood and the levels of sIL-6R in the sera: in sera of three patients with lymphocyte counts of 60 000 cells/μl or more, the levels of sIL-6R were 74 ng/ml ± 25 ng/ml whereas when the counts were below 60 000 the mean levels were 33.4 ng/ml ± 14 ng/ml (seven patients). The difference between the two groups was significant at $p = 0.01$.

### Discussion

IL-6 is a cytokine controlling later stages of B cell maturation and Ig production. It has been found in supernatants from cultures of freshly isolated B cells from hypergammaglobulinaemic patients. Rickmann *et al.*[20] prepared antibodies against IL-6 which suppressed spontaneous and *Staphylococcus aureus* Cowan 1 or IL-2 induced production *in vitro*.

We found IL-6 and sIL-6R in the supernatants of unstimulated B-CLL B cell cultures so corroberating the findings of Biondi *et al.*[21] that B-CLL lymphocytes produce and secrete IL-6 *in vitro* in the absence of specific stimulation. IL-6 was expressed on the membrane of some but not all B-CLL B cells, which were $CD14^-$ or $CD14^+$ but always $CD5^+$.

In the presence of PMA, B-CLL B lymphocytes released less IL-6 in the supernatants of cultures already after 2 h of incubation, whereas sIL-6R levels increased markedly by comparison with unstimulated controls. A similar but weaker effect on sIL-6R release was observed in cultures of normal B lymphocytes in the presence of PMA. In a recent study, Mullberg *et al.*[22] showed that the ligand binding subunit gp80 of human IL-6R was shed by transfected COS-7 cells. The shedding was greatly increased by exposing the cells to PMA. PMA treatment induced protein kinase C (PKC) that regulated shedding of sIL-6R.[22] These data accord well with our findings that treatment with PMA markedly increased the levels of sIL-6R in culture supernatants. The effect of PWM was variable and not significant. A possible explanation for the difference in effects between the two stimulants could be due to the property of PMA to promote mainly terminal differentiation of B cells for which IL-6 is necessary[23] and thus, is utilized by the cells, while in the same time sIL-6R is shed into the culture medium. PWM stimulates mainly proliferation of B lymphocytes which is probably less dependent on IL-6.[24] Two types of possible functions of sIL-6R were postulated: an antagonistic activity or an agonistic mode of action. We did not attempt to define what is the most likely possibility in our experimental system.

No significant difference was observed between the levels of IL-6 and sIL-6R in the supernatants from purified and nonpurified B-CLL B cell cultures, thus indicating that the main contribution was made by B lymphocytes. However, a trend to increased levels of IL-6 in cultures of not purified B-CLL B lymphocytes might be due to additional secretion of IL-6 by residual T cells.

IL-6 was not detected in culture supernatants of unstimulated or stimulated normal B lymphocytes. The difference between the results obtained with B-CLL versus normal B lymphocytes could be due to the fact that B-CLL B cells are stimulated B cells and as such they secrete IL-6 into the culture medium. Reaction of IL-6 with IL-6R upregulated by PMA, could result in shedding of sIL-6R into the culture supernatants. This will occur to a far lesser extent with normal B cells because the level of IL-6 in the culture supernatants was much lower.

The levels of IL-6 in sera of normal subjects was below the limit of detection ($<60$ pg/ml). Surprisingly this was also the case with sera from B-CLL patients in spite of the high levels of IL-6 detected in cultures of B-CLL B cells, although the number of B cells in the peripheral blood of B-CLL patients was from 20 to 60 times higher than in normal subjects. A plausible explanation for this discrepancy could be that IL-6 is taken up and utilized by various cells in the body while at the same time sIL-6R is probably secreted into the blood possibly after reaction with IL-6, thus allowing detection of measurable quantities in the serum. It was recently shown that sIL-6R in body fluids is biologically active.[25] Our finding of direct correlation between the levels of sIL-6R in the serum and the number of circulating B-CLL B lymphocytes is consistent with the view that increase in sIL-6R levels is driven by IL-6.

The small number of B-CLL patients belonging to the more advanced stages of Rai classification 3 and 4, does not allow any clear correlation between the stages of B-CLL and the serum IL-6 and sIL-6R levels. However, B lymphocytes from two cases with Rai stage 3 produced less than 5 ng/ml of IL-6 in cultures, whereas most cases with Rai stage 4 produced more than 10 ng/ml.

### References

1. Hirano T, Yasukawa K, Harada H. Complementary DNA for a novel human interleukin (BSF-2) that induces B lymphocytes to produce immunoglobulins. *Nature (London)* 1986; **324**: 73–76.
2. Yoshizaki K, Nakawa K, *et al.* Isolation and characterization of B cell lymphoid line. *J Immunol* 1984; **132**: 2948–2954.
3. Matsuzaki N, Fumigata S, Kameda T, *et al. In vitro* and *in vivo* production of interleukin-6 by fetal mononuclear cells. *Clin Immunol Immunopathol* 1990; **55**: 305–314.
4. Horii Y, Maraguchi A, Suematu A, *et al.* Regulation of BSF-/IL-6 production by human mononuclear cells. *J Immunol* 1988; **141**: 1529–1535.
5. Kishimoto T. The biology of interleukin-6. *Blood* 1989; **74**: 1–10.
6. Nordan RP, Potter M. A macrophage-derived factor required by plasmacytoma for survival and proliferation *in vitro*. *Science* 1986; **233**: 566–569.
7. German RD, Jacobs KA, Clark SC, Raulet DH. B-cell stimulating factor 2 functions as a second signal for interleukin 2 production. *Proc Natl Acad Sci USA* 1987; **84**: 7629–7621.
8. Navarro S, Debili N, LeCouedic JP. Interleukin-6 and its receptor are expressed by human megakaryocyte. *Blood* 1991; **77**: 461–471.
9. Kishimoto T, Akira S, Taga T. IL-6 receptor and mechanism of signal transduction. *Int J Immunopharmac* 1992; **14**: 431–438.
10. Novick D, Englemann H, Wallach D, *et al.* Soluble cytokine receptors are present in normal human urine. *J Exp Med* 1989; **170**: 1409–1414.
11. Novick D, Cohen B, Rubinstein M. Soluble interferon receptor molecules are present in body fluids. *FEBS* 1992; **314**: 445–448.
12. Engelman H, Novick D, Wallach D. Two human-necrosis factor binding proteins purified from human urine. *J Biol Chem* 1989; **265**: 1531–1536.
13. Maliszewski CR, Fanslow WC. Soluble receptor for IL-1 and IL-4: biological activity and therapeutic potential. *Trends Biotechnol* 1990; **8**: 324–329.
14. Novick D, Engelman H, Revel M, *et al.* Monoclonal antibodies to the soluble human IL-6 receptor. *Hybridoma* 1991; **10**: 137–145.
15. Rai KR, Mousserat M. Clinical staging of chronic lymphocytic leukemia. *Blood* 1975; **46**: 219–223.
16. Farcas R, Manor Y, Klajman A. Long term cultures of chronic lymphocytic leukemia B cells generate B suppressor cells. *Clin Immunol Immunopathol* 1987; **42**: 171–182.
17. Farcas R, Ben-Efraim S, Manor Y, Klajman A. Appearance of CD8 marker on B-CLL cells in long term cultures. *Cancer Immunol Immunoth* 1991; **34**: 181–185.
18. Novick D, Eshar Z, Revel M, Mory Y. Monoclonal antibodies for affinity purification of IL-6/IFN-β and for neuralization of HGF activity. *Hybridoma* 1989; **8**: 561–566.
19. Novick D, Revel M, Rubinstein M. High levels of soluble IL-6 receptor and low levels of IL-6 in normal human serum. *Cytokine* 1991; **3**: 4.
20. Rickmann P, D'Allessandro F, Nordan RP. IL-6 and tumor necrosis factor-α. *J Immunol* 1991; **146**: 3462–3466.

21. Biondi A, Rossi V, Bassan R. Constitutive expression of the interleukin-6 gene in chronic lymphocytic leukemia. *Blood* 1989; **73**: 1279–1284.
22. Mullberg J, Scholltink H, Stoyan T. The soluble interleukin-6 receptor is generated by shedding. *Eur J Immunol* 1993; **23**: 473–480.
23. Hirano T. Interleukin-6 and its relation to inflammation and disease. *Clin Immunol Immunopath* 1992; **62**: 560–565.
24. Levi Y, Fermand JP, Brouet J. Differential effects of low and high concentrations of interleukin-6 on human B cells. *Eur J Immunol* 1990; **20**: 2389–2993.
25. Novick D, Shulmann LM, Chen L, Revel M. Enhancement of Il-6 cytostatic effect of human breast carcinoma cells by sIL-6R from urine and reversion by monoclonal antibody. *Cytokine* 1992; **4**: 6–11.

**Received 6 January 1997;
accepted in revised form 6 February 1997**
